# Tripeptide IRW Improves AMPK/eNOS Signaling Pathway via Activating ACE2 in the Aorta of High-Fat-Diet-Fed C57BL/6 Mice

**DOI:** 10.3390/biology12040556

**Published:** 2023-04-06

**Authors:** Fatemeh Ashkar, Khushwant S. Bhullar, Xu Jiang, Jianping Wu

**Affiliations:** Department of Agricultural Food and Nutritional Science, University of Alberta, Edmonton, AB T6G 2R3, Canada

**Keywords:** IRW, insulin resistance, GLUT4, eNOS, ACE2, peptides

## Abstract

**Simple Summary:**

Metabolic syndrome refers to a cluster of various risk factors commonly associated with cardiovascular diseases and diabetes. Upregulation of angiotensin-converting enzyme 2 (ACE2), a key member of the renin–angiotensin system (RAS), is in general protective against cardiovascular diseases and diabetes. Peptide IRW was previously shown to upregulate ACE2 in spontaneously hypertensive rats, but not the skeletal muscle ACE2 in a high-fat-diet (HFD)-induced insulin-resistant mouse model. The study aims to investigate the regulatory role of peptide IRW on aortic ACE2 and its associated signaling pathways in HFD-induced insulin-resistant mice. Our results indicated that upregulation of aortic ACE2 by peptide IRW in HFD-induced insulin-resistant mice is responsible for the activation of the pathways associated with vasodilation of blood vessels, which might play a role in improving insulin resistance and glucose metabolism.

**Abstract:**

This study aims to investigate the effect of tripeptide IRW on the local renin–angiotensin system (RAS), particularly angiotensin-converting enzyme 2 (ACE2), and their association with signaling pathways in the aorta of a high-fat-diet (HFD)-induced insulin-resistant mouse model. C57BL/6 mice were fed HFD (45% of the total calories) for six weeks, and then IRW was added to the diet (45 mg/kg body weight (BW)) for another eight weeks. ACE2 mRNA expression and protein level(s) were increased (*p* < 0.05), while angiotensin II receptor (AT1R) and angiotensin-converting enzyme (ACE) protein abundance was significantly reduced (*p* < 0.05) in the aorta of HFD mice treated by IRW. IRW supplementation also improved glucose transporter 4 (GLUT4) abundance (*p* < 0.05) alongside AMP-activated protein kinase (AMPK) (*p* < 0.05), Sirtuin 1 (SIRT1) (*p* < 0.05), and endothelial nitric oxide synthase (eNOS) (*p* < 0.05) expression. IRW downregulated the levels of endothelin 1 (ET-1) and p38 mitogen-activated protein kinases (p38 MAPK, *p* < 0.05). Furthermore, the levels of AMPK and eNOS in vascular smooth muscle cells (VSMCs) were significantly reduced in ACE2 knockdown cells treated with or without IRW (*p* < 0.01). In conclusion, this study provided new evidence of the regulatory role of IRW on the aortic ACE2 against metabolic syndrome (MetS) in an HFD-induced insulin-resistant model.

## 1. Introduction

Metabolic syndrome (MetS) is the combination of risk factors associated with cardiovascular disease (CVD) and type 2 diabetes (T2D), including hypertension, dyslipidemia, impaired glucose tolerance, and obesity [1]. Visceral obesity and insulin resistance, the main pathophysiologic features of MetS, are strongly associated with high blood pressure [2]. A key additional component of insulin resistance is the renin–angiotensin system (RAS), largely known for its role in blood pressure regulation [3]. The major biologically active stress component generated by this system is angiotensin II (Ang II) [4]. While angiotensin-converting enzyme (ACE) is responsible for the generation of Ang II, angiotensin-converting enzyme 2 (ACE2) is involved in the conversion of Ang II into angiotensin (Ang 1-7). Ang 1-7 displays anti-inflammatory and anti-hypertensive properties, which antagonize Ang II’s harmful vascular impacts [5,6]. The expression of local RAS components, especially the Ang II–angiotensin II receptor (AT1R) axis, is enhanced by hyperglycemia, hypertension, hyperinsulinemia, and obesity associated with the metabolic syndrome [7]. ACE2 was observed to reduce insulin resistance by decreasing the production of Ang II, potentially providing a therapeutic approach for the management of MetS [8]. 

Bioactive food components, such as bioactive peptides, have attracted the interest of global food scientists for their potential in the prevention and treatment of MetS [9]. IRW (Ile-Arg-Trp), a bioactive tripeptide, was identified and characterized from egg white protein ovotransferrin as an activator of ACE2 in hypertensive animals [8,10]. Thus, IRW could shift the balance from vasoconstrictor and inflammatory actions of Ang II towards cytoprotective and vasorelaxant effects via ACE2 activation [8,10,11]. Recently, studies showed that the expressions of glucose transporter 4 (GLUT4) and glucose uptake were reduced in hypertension and diabetes [12,13]. Moreover, Ang II-mediated hypertension mouse models showed lower GLUT4 expression and glucose absorption in the mice aorta [14], further supporting an underlying interplay between hypertension and T2D. 

In our previous high-fat diet (HFD) model study, we noticed no significant difference in ACE2 expression in skeletal muscle of HFD mice treated with or without IRW [15], refuting a role of local ACE2 activation. However, it should be noted that ACE2 is significantly upregulated in mesenteric and aorta arteries of spontaneously hypertensive rats (SHRs) [12], which prompted us to further conduct a study to investigate the role of aorta ACE2 in response to IRW treatment. Although skeletal muscle is the major site of glucose metabolism, vascular function, especially endothelial dysfunction, plays a key role in the pathogenesis of hypertension [16] as well as insulin resistance and T2D [17]. HFD is widely used to induce obesity and insulin resistance in preclinical animal studies [18] and is also a key contributor to endothelial dysfunction in vivo [19]. HFD can induce chronic inflammation via oxidative stress that disrupts vascular structure and function, resulting in endothelial and physiological dysfunction, and initiate the onset and development of metabolic syndrome [20]. Therefore, in order to gain further insight into the regulatory role of aorta ACE2 in the insulin signaling pathway and endothelial function, this study aims to investigate the effect of IRW on RAS, particularly ACE2, and their association with signaling pathways in the aorta of an HFD-induced obese mouse model.

## 2. Materials and Methods

### 2.1. Chemicals and Reagents

Dulbecco’s phosphate-buffered saline, Dulbecco’s modified Eagle medium (DMEM), Opti-MEM I Reduced Serum Medium, fetal bovine serum (FBS), and antibiotics penicillin and streptomycin were purchased from Gibco/Invitrogen (Carlsbad, CA, USA). Lipofectamine 2000 Transfection Reagent and interference RNA (siRNA; 10 µm/L) were acquired from Thermo Fisher Scientific (Waltham, MA, USA). Triton-X-100 was procured from VWR International (West Chester, PA, USA). The synthesized tripeptide IRW (>99.8% purity) was purchased from Genscript (Piscataway, NJ, USA). All other reagents and chemicals of analytical grade were provided by Sigma-Aldrich (St. Louis, MO, USA).

### 2.2. Animal Model Study

Animal Care and Use Committee of the University of Alberta approved our study (protocol# 1402) in line with the Canadian Council on Animal Cares standard. Eighteen male C57BL/6 mice, aged 4 weeks, were obtained from Charles River Canada and given ad libitum access to food and water for one week. The environment was regulated with a 12:12 h light–dark cycle, with 60% humidity and 23 °C temperature. Six of the mice were fed a low-fat diet (10% kcal from fat) and the rest were provided with a HFD (45% kcal from fat) over 6 weeks. Following this period, the mice were randomly separated into three groups: low-fat diet, HFD, and HFD plus IRW (45 mg/kg BW/d). All three groups had *ad libitum* access to food and water for 8 weeks, with the HFD plus IRW group additionally receiving IRW (45 mg/kg BW/d) for the duration.

### 2.3. Tissue Collection

Before euthanasia, all animals were fasted for 16 h before being injected intraperitoneally with insulin (2 IU/kg BW) to stimulate insulin signaling. Blood was collected via cardiac puncture after animals were euthanized with CO_2_. In order to obtain plasma, blood was centrifuged at 3000× *g* for 15 min and stored at −80 °C. Aortic vascular smooth muscle tissues were collected, washed with ice-cold saline, weighed, immediately frozen in liquid nitrogen, and stored at −80 °C for further analysis.

### 2.4. Protein Extraction and Western Blotting

Tissue proteins were extracted by protein extraction buffer (20 mm Tris, 5 mM EDTA, 10 mM Na_4_P_2_O_7_, 100 mM sodium fluoride, and 1% NP-40) containing a 1% (*v*/*v*) protease inhibitor cocktail (Thermo Fisher Scientific, Waltham, MA, USA). Then, the homogenates were centrifuged at 15,000× *g* for 15 min at 4 °C. Protein concentrations in the supernatants were determined by bicinchoninic acid (BCA) assay (Thermo Fisher Scientific). Samples (calibrated to the same protein mass) were loaded on a 9% separating gel and transferred to a nitrocellulose membrane (diameter 0.45 µm; Bio-Rad, Montreal, QC, Canada) for incubation with antibodies of ACE (Abcam, Toronto, ON, Canada), ACE2 (Abcam, Toronto, ON, Canada), AT1R (Novus biologicals, Oakville, ON, Canada), endothelial nitric oxide synthase (eNOS; BD Biosciences, San Jose, CA, USA), phospho-eNOS (p-eNOS Ser1177, Abcam), phospho AMP-activated protein kinase (p-AMPKα Thr172, cs2535), phospho p38 mitogen-activated protein kinases (p38 MAPK, NOVUS Biologicals), phospho extracellular signal-regulated kinase (p-ERK1/2, Cell Signaling Technology, Whitby, ON, Canada), endothelin 1 (ET-1, Abcam), Sirtuin 1 (SIRT1; 9475, Cell signaling Technology, Danvers, MA, USA), peroxisome proliferator-activated receptor gamma (PPARγ, cs2430), and GLUT4 (Abcam, Toronto, ON, Canada). All bands were normalized to glyceraldehyde 3-phosphate dehydrogenase (GAPDH; Abcam). Goat-anti-rabbit IRDye 680 RD or Donkey-anti-mouse 800 CW secondary antibodies were used to visualize the bands in a Licor Odyssey Bio Imager, with the fluorescence signal quantified using Image Studio Lite 5.2 (Licor Biosciences, Lincoln, NE, USA).

### 2.5. Cell Culture

In this study, the A7r5 cell line (ATCC CRL1444, Manassas, VA, USA) was used between passages 8 and 11. The cell culture was performed in 12-well plate(s) in a humidified atmosphere with 95% air/5% CO_2_ at 37 °C. The cells were grown in DMEM medium supplemented with 10% FBS and antibiotics (penicillin–streptomycin and streptomycin) at 5% CO_2_ level. Once reaching ∼80% confluence, the cell culture media were replaced with DMEM supplemented with 1% FBS and antibiotics and then treated with 50 µM IRW for 24 h to measure protein expressions of eNOS and AMPK in the cells treated with or without IRW.

### 2.6. SiRNA Transfection

To silence ACE2, siRNA and lipofectamine 2000 transfection reagent were used in A7r5 cells. The cells were placed overnight in non-antibiotic DMEM with 10% FBS after reaching 50% confluence. For ACE2 knockdown, the non-antibiotic DMEM for cell culture media was changed to serum-reduced Opti-MEM media. Each targeting well was treated by ACE2 siRNA (80 pmol) containing 0.8 µL of transfection reagent. After 6 h of incubation, non-antibiotic DMEM with 10% FBS was replaced with serum-reduced Opti-MEM media for 24 h. The efficiency of knockdown was at least 70%. After 24 h, cells were placed in the non-antibiotic medium (DMEM + 1% FBS) and then treated with 50 µM of IRW to measure protein expressions of eNOS and AMPK in the cells treated with ACE2 siRNA.

### 2.7. RT-PCR

Total RNA was isolated from the aorta of mice with TRIzol solution and 1 μg of total RNA was then used to synthesize cDNA via the High-Capacity cDNA Reverse Transcription Kit (Thermo Fisher Scientific). The expression of targeted genes was measured with real-time qPCR, using GAPDH as an internal control, and the MIQE guidelines for qPCR were followed for all experiments and analyses.

### 2.8. Statistics

The results are presented as mean ± SEM of a minimum of three independent experiments. Data were analyzed by one-way analysis of variance (ANOVA) coupled with Dennett’s test by Prism 6 statistical software (GraphPad Software, San Diego, CA, USA). *p* < 0.05 was considered statistically significant.

## 3. Results

### 3.1. IRW Treatment Upregulated ACE2 and Diminished ACE and AT1R Expression in the Aorta

**Figure 1:**
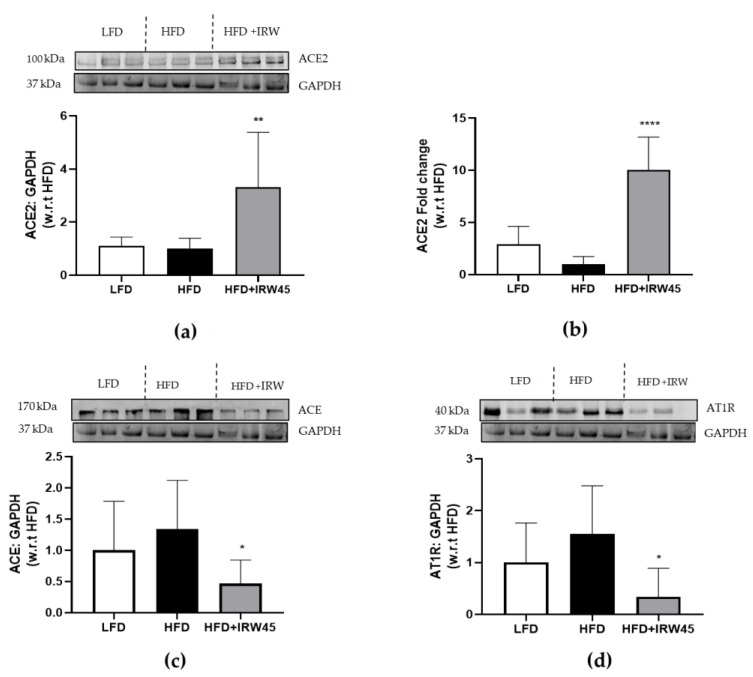
Effect of IRW on protein and RNA expression of ACE2 and protein expression of ACE and AT1R in aorta of HFD mice. (**a**) Quantification and Western blots of ACE2. (**b**) q-PCR quantification of ACE2. Quantification and Western blots of (**c**) ACE and (**d**) AT1R. ACE2, ACE, and AT1R were normalized to GAPDH. Data expressed as mean ± SEM of n = 6 mice. *, *p* < 0.05, **, *p* < 0.01, and ****, *p* < 0.0001 versus HFD. (Fold change with regard to (w.r.t) HFD).

ACE2-Ang-(1-7)-Mas receptor (MasR) plays an imperative role in inhibiting oxidation, proliferation, and inflammation in vascular smooth muscle cells (VSMCs). To explore whether IRW could improve ACE2 in the aorta of HFD mice, both protein and RNA levels of ACE2 were examined following treatment of IRW (45 mg/kg body weight) for 8 weeks. Protein and RNA levels of ACE2 were significantly increased in the IRW group compared to the HFD (*p* < 0.01) (Figure 1). Ang II exerts detrimental effects mainly through AT1R (11); the levels of ACE and AT1R were also examined. Our results showed that IRW significantly decreased ACE and AT1R levels in IRW-treated mice compared to the HFD (*p* < 0.05) (Figure 1).

### 3.2. IRW Enhanced AMPK/SIRT1/eNOS Cascade in Aorta of HFD Mice via Aortic ACE2 Activation

**Figure 2:**
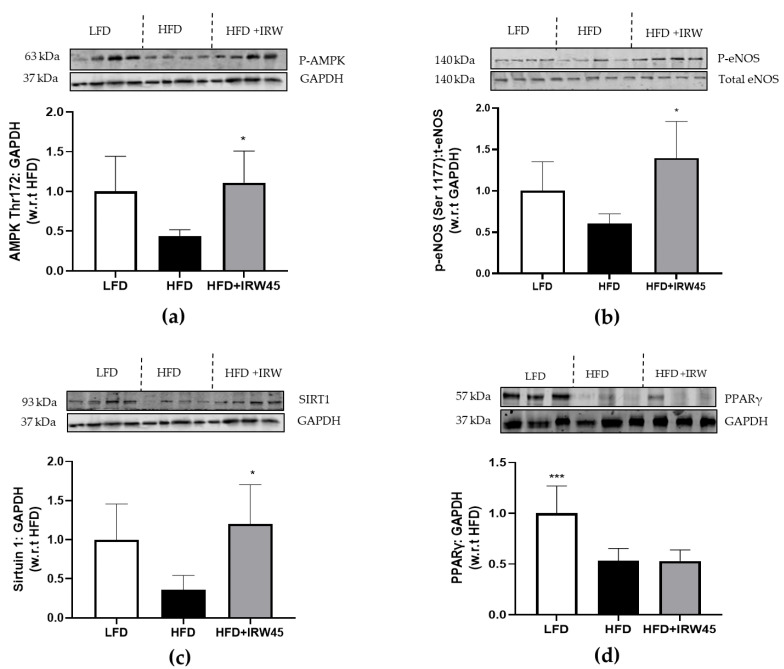
Effect of IRW on expression of p-AMPK, p-eNOS, Sirtuin1, and PPARγ in aorta of HFD mice. Quantification and Western blots of (**a**) p-AMPK, (**b**) p-eNOS, (**c**) Sirtuin 1, and (**d**) PPARγ. P-AMPK, Sirtuin1, and PPARγ were normalized to GAPDH. p-eNOS was normalized to Total eNOS. Data expressed as mean ± SEM of *n* = 4 for p-eNOS, Sirtin 1, and p-AMPK, and *n* = 6 for PPARγ. *, *p* < 0.05, ***, *p* < 0.001 versus HFD. (Fold change with regard to (w.r.t) HFD).

**Figure 3:**
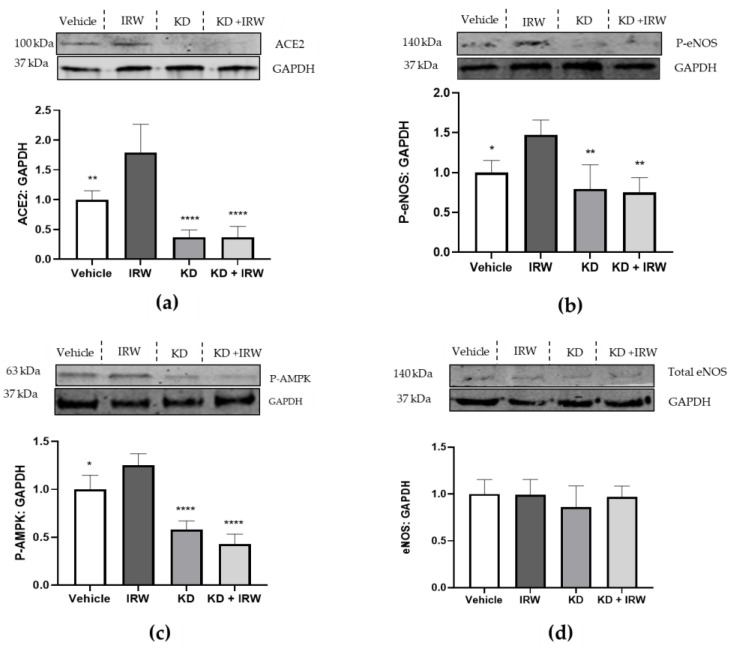
Effect of IRW on expression of ACE2, p-eNOS, p- AMPK, and Total eNOS in vehicle, IRW-treated, and ACE2 knockdown (KD) VSMCs. Quantification and Western blots of (**a**) ACE2, (**b**) p-eNOS, (**c**) p-AMPK, and (**d**) Total eNOS. ACE2, p-AMPK, p-AMPK, and Total eNOS were normalized to GAPDH. Data expressed as mean ± SEM of four independent experiments. *, *p* < 0.05, **, *p* < 0.01, and ****, *p* < 0.0001 versus IRW-treated group.

We previously showed that IRW treatment significantly improved endothelial function in SHR via ACE2 activation [19,21,22]. There is strong evidence that endothelial dysfunction plays a causal role in the development of insulin resistance and the progression of diabetes mellitus [9,23]. Thus, biomarkers associated with endothelial function such as AMPK, PPARγ, SIRT1, and eNOS were detected. In the next experiments, we examined whether IRW treatment induces AMPK/SIRT1/eNOS cascade in the aorta of HFD mice. AMPK and SIRT1 both regulate each other and share many common target molecules in metabolic syndrome [24]. HFD feeding reduced PPARγ expression (*p* < 0.01). Treatment with IRW significantly elevated the p-AMPK (The172), SIRT1, and p-eNOS (Ser1177) protein expression (*p* < 0.05); however, no significant changes were observed in the total PPARγ abundance (Figure 2). The correlation of ACE2 with AMPK and eNOS was further evaluated by ACE2 knockdown experiment in A7r5 cells. Interestingly, ACE2 knockdown significantly decreased the expression of p-AMPK and p-eNOS in the VSMCs (*p* < 0.01) (Figure 3). Overall, these results show that p-AMPK and p-eNOS are positively regulated by ACE2 but negatively associated with insulin resistance.

### 3.3. IRW Improved GLUT4 in Aorta of HFD Mice

**Figure 4:**
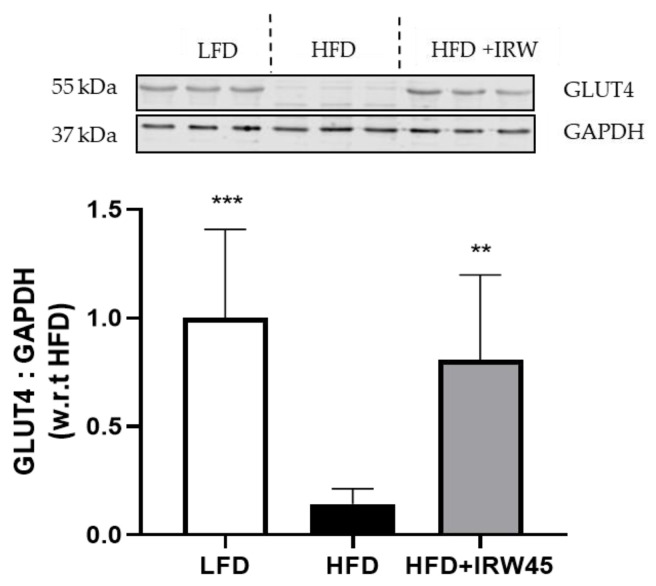
Effect of IRW on expression of GLUT4 in aorta of HFD mice. Quantification and Western blots of GLUT4. GLUT4 was normalized to GAPDH. Data expressed as mean ± SEM of n = 6 mice. **, *p* < 0.01 and ***, *p* < 0.001 versus HFD. (Fold change with regard to (w.r.t) HFD).

GLUT4 is an important glucose transporter to uptake glucose in skeletal muscle [14]. Moreover, the effectiveness of GLUT4 is reliant on the activity of AMPK in skeletal muscle [25]. As shown in Figure 4, the protein level of GLUT4 was reduced significantly in the aorta of HFD, whereas GLUT 4 translocation to the plasma membrane was higher in the IRW group (*p* < 0.01) compared to the HFD group.

### 3.4. IRW Downregulated ET1/MAPK Pathway

**Figure 5:**
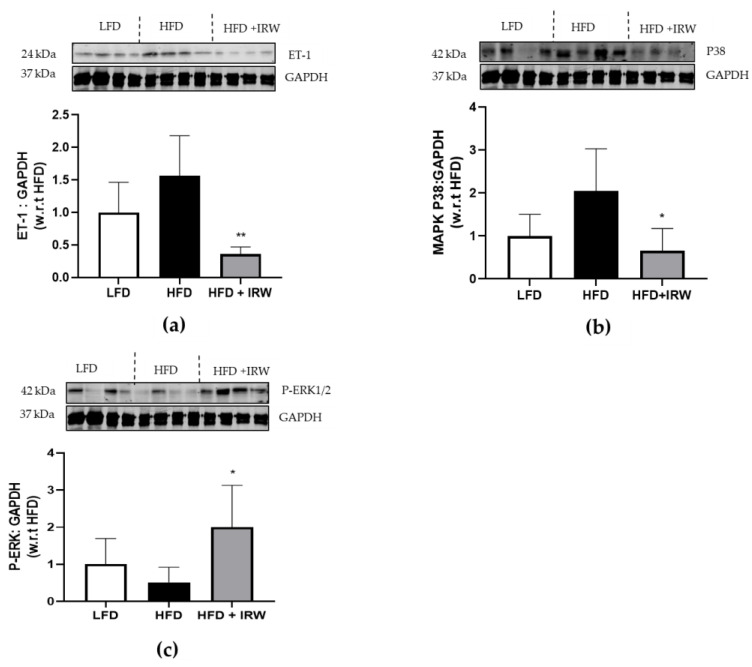
Effect of IRW on expression of ET-1, MAPK P38, and P-ERK1/2 in aorta of HFD mice. (**a**) Quantification and Western blots of ET-1, (**b**) MAPK P38, and (**c**) p-ERK. ET-1, MAPK P38, and p-ERK were normalized to GAPDH. Data expressed as mean ± SEM of n = 4 mice. *, *p* < 0.05, **, *p* < 0.01 versus HFD. (Fold change with regard to (w.r.t) HFD).

MAPK signaling pathways are activated in VSMCs by ET-1, which is a mediator of Ang II-mediated signaling [26,27]. Next, signal transduction of ET-1/MAPK in the aorta of HFD was investigated. IRW treatment in HFD significantly decreased the levels of ET-1 (*p* < 0.01) and p38 MAPK (*p* < 0.05), which are key members of migration and proliferation remodeling of the vascular system. However, the IRW group showed a significant increase in p-ERK expression compared to the HFD group (*p* < 0.05) (Figure 5). This result suggested that IRW at least partially was involved in the modulatory effects of the Ang II–AT1R axis on ET-1/MAPK (P38) in the aorta of mice.

## 4. Discussion

MetS has attracted great attention because the prevalence of obesity and related chronic diseases have increased globally [28,29]. Recent findings have revealed that approximately 25% of the global population suffers from metabolic syndrome [30]. It is possible to treat these diseases with synthetic drugs; however, this can lead to some unfavorable consequences. The tripeptide IRW has been shown to improve glucose tolerance and lower fasting blood glucose and insulin concentrations in the skeletal muscle of mice fed HFD via insulin-dependent signaling and independent pathways [15]. Moreover, the anti-inflammatory and antioxidant activities of IRW, along with its ability to upregulate eNOS and nitric oxide (NO), support its ability to mediate vasorelaxation of blood vessels [19]. In the current study, we provided evidence that IRW could enhance ACE2 level in the aorta of HFD mice, proposing that the aorta might be a specific target of IRW to affect ACE2 activity in MetS. Additionally, ACE2 knockdown caused a marked attenuation of p-AMPK and p-eNOS markers in A7r5 cells, indicating the regulatory role of ACE2 on AMPK and eNOS. These findings suggested that IRW with an increase in ACE2 level and mitigation of the AT1R receptor via multiple signaling pathways including ACE2/AMPK/SIRT1/eNOS, ACE2/AMPK/GLUT4, and ATIR/ET-1/P38 MAPK in the mouse aorta might be a therapeutic agent in MetS. This study was the first to demonstrate that IRW could be linked to MetS through a novel pathway characterized by aortic ACE2 activation.

The RAS has a significant impact on the initiation and progression of insulin resistance [31]. There is evidence that metabolic complications, such as diabetes and obesity, are associated with the upregulation of RAS components such as angiotensinogen, ACE, and AT1R [32]. ACE2 has been found to counteract the effects of Ang II, resulting in lower blood pressure and a decreased risk of developing CVD [33]. Despite no effect of IRW on ACE2 and ACE levels of skeletal muscle being observed on the insulin resistance model [15], we found a decrease in ACE and AT1R protein expression in the aorta of HFD mice treated with IRW, suggesting the modulatory role of IRW on aortic RAS, which is related to MetS. On the other hand, our previous study showed that oral intake of IRW improved the amount and activity of ACE2 in the blood, as well as the amount of ACE2 protein in the aorta of SHR rats [5,10]. Similarly, IRW increased both ACE2 RNA level and protein expression in the aorta of the insulin-resistant mouse model. This could be attributed to the fact that the genes of the RAS elements were mainly expressed in the organs, such as the heart, brain, kidneys, and aorta [34]. Therefore, ACE2 might provide novel insight into the molecular mechanism associated with MetS.

AMPK activation was recently proposed as a potential therapeutic target for the prevention and amelioration of insulin resistance and T2D [35,36]. Our prior study demonstrated an increase in p-AMPK and PPARγ in the skeletal muscle of HFD mice treated with IRW, but ACE2 was unaffected, suggesting that IRW could potentially improve glucose metabolism independently of ACE2 in the skeletal muscle of HFD mice [15]. Moreover, it was observed that the endothelial-dependent vasodilatory response is impaired in insulin resistance [37]. The endothelial AMPK/eNOS pathway accounted for adequate endothelial function in the whole aorta [38]. Activation of AMPK in endothelial cells by metformin led to the phosphorylation of eNOS at Ser1177, resulting in the production of NO and subsequent dilation of the mice’s aorta [25,39]. NO is a critical player in vascular homeostasis and maintenance of blood pressure [40], and the development and progression of diabetes mellitus are related to the alteration in eNOS expression and activity [23,41,42]. Furthermore, upon treatment with rosiglitazone, an insulin sensitizer, the diabetic mouse indicated an increase in the release of adiponectin, which activated AMPK/eNOS signaling pathways in the aorta, subsequently reducing oxidative stress and amplifying NO bioavailability [43]. This allowed for an improvement in the mouse’s endothelial function due to PPARγ activation [43]. Our study also indicated the ability of IRW in increasing p-AMPK and p-eNOS in the aorta of HFD mice. These results are in accordance with our previous study that IRW led to a rise in p-eNOS and NO-mediated dilation of the mesenteric arteries of SHRs [19]. Although the protein expression of PPARγ decreased in HFD mice, IRW treatment did not exhibit any effect on PPARγ protein abundance, suggesting that IRW might only be able to regulate PPARγ in the skeletal muscle of the mice with HFD-induced metabolic syndrome. On the other hand, our study showed reduced levels of p-AMPK and p-eNOS in ACE2 knockout VSMCs, which is in accordance with a recent study indicating that ACE2 deficiency caused a decrease in cardiac p-AMPK levels in knockout rats [44]. Thus, our results suggested that IRW, particularly affecting aortic ACE2, could play a crucial role in the MetS by modulating the ACE2/AMPK/eNOS pathway.

SIRT1 plays an important role in endothelial biology as well. Deacetylating eNOS by SIRT1 prevents oxidative stress-induced endothelial senescence and increases endothelial-dependent vasodilation [45,46]. Additionally, SIRT1 and AMPK work together to regulate cell stress and energy balance and protect the cardiovascular system [47]. According to previous research, a deficiency in SIRT1 and p-AMPK has been linked to endothelial dysfunction [47]. Our findings suggested that IRW was able to increase AMPK levels, which was regulated by aortic ACE2, and subsequently led to an increase in SIRT1 expression in HFD mice. This could potentially influence MetS and associated pathways.

Our recent study revealed that activation of AMPK without affecting ACE2 promoted GLUT4 translocation to the cell membrane, which ultimately stimulated glucose uptake in skeletal muscle [15]. According to the inhibition of basal glucose uptake in the mice aortas by GLUT4 antagonist (indinavir), it was demonstrated that ~50% of basal glucose uptake in VSMCs was mediated by GLUT4 in vivo [14]. In addition, it was shown that VSMC GLUT4 levels and glucose uptake were decreased in rats with diabetes mellitus [12]. Similarly, we observed the improvement of membrane localization of GLUT4 in the aorta of HFD mice treated with IRW. Additionally, we observed the regulatory role of ACE2 on AMPK, proposing that the ACE2/AMPK/GLUT4 signaling pathway in the aorta might play a role in regulating MetS. Although evidence demonstrated that AMPK had a positive influence on GLUT4, no proof of modulating GLUT4 by AMPK was reported in VSMCs. The exact mechanism by which IRW enhances GLUT4 in VSMCs with and without aortic ACE2 expression remains unknown; thus, further research is required to explore other possible pathways.

ET is a potent vasoconstrictor and stimulates the renin–angiotensin–aldosterone system. The ET-1 receptor being triggered causes the MAPK cascade to occur, which is a key signaling event in endothelial function. MAPKs are serine/threonine protein kinases playing a significant role in mediating Ang II-induced signaling in VSMCs [22,27]. Hyperinsulinemia could trigger MAPK pathways, leading to reduced NO production and higher ET-1 release, which are linked to impaired endothelial function [16,48]. It was shown that expressions of p-ERK and several upstream signaling p38 proteins in the MAPK pathway in the HFD renal tissues were elevated significantly compared to the normal group [49]. Previously, IRW reduced the phosphorylation of p38 when VSMCs were stimulated with Ang II, implying that IRW could have an important function in regulating MAPKs through the AngII/AT1R pathway [11]. However, IRW treatment did not prevent ERK1/2 MAPK phosphorylation even with an increase in Ang (1-7) in the aorta of SHRs [5]. Consistent with these results, the current study demonstrated that HFD only induced an increase in ET-1 and P38 MAPK in the aorta, which could be countered by IRW treatment in vivo. No significant decrease in ERK1/2 expression was observed after IRW treatment in the aorta of HFD mice, suggesting that further investigation into the endothelial function related to MetS is required. Further functional studies in isolated mice aortic rings, histopathology of the VSMCs in the aorta, and immunohistochemistry to identify protein nuclear localization should be considered to provide strong evidence for the potential use of IRW as a treatment.

## 5. Conclusions

This research indicated that ACE2 in the aorta of HFD obese mice might play an important role in maintaining the ACE2/AMPK/eNOS signaling pathways. Therefore, aortic ACE2 might be a potential focus of IRW to improve glucose uptake and vasodilation of blood vessels in MetS. Moreover, IRW might play a crucial role in the suppression of AT1R/ET-1/ p38 MAPK signaling pathways related to MetS in vitro and in vivo models. Overall, our study is the first to link the effect of bioactive peptides on MetS with the local RAS, in particular, ACE2 in the aorta of HFD mice. Furthermore, our data suggest that IRW holds strong potential for modulating metabolic complications by aortic ACE2 regulation in vivo. Thus, this makes IRW worthy of further investigation as a therapeutic agent for MetS in the aorta.

## Data Availability

Not applicable.
